# Insights into the pathological basis of dementia from population‐based neuropathology studies

**DOI:** 10.1111/nan.12923

**Published:** 2023-08-06

**Authors:** Stephen B. Wharton, Julie E. Simpson, Paul G. Ince, Connor D. Richardson, Richard Merrick, Fiona E. Matthews, Carol Brayne, CFAS

**Affiliations:** ^1^ Sheffield Institute for Translational Neuroscience University of Sheffield Sheffield UK; ^2^ Population Health Sciences Institute Newcastle University Sheffield UK; ^3^ Cambridge Public Health, School of Clinical Medicine University of Cambridge Sheffield UK

**Keywords:** Alzheimer's disease, dementia neuropathology, epidemiological neuropathology, Lewy body disease, population‐representative neuropathology, vascular dementia

## Abstract

The epidemiological neuropathology perspective of population and community‐based studies allows unbiased assessment of the prevalence of various pathologies and their relationships to late‐life dementia. In addition, this approach provides complementary insights to conventional case–control studies, which tend to be more representative of a younger clinical cohort. The Cognitive Function and Ageing Study (CFAS) is a longitudinal study of cognitive impairment and frailty in the general United Kingdom population. In this review, we provide an overview of the major findings from CFAS, alongside other studies, which have demonstrated a high prevalence of pathology in the ageing brain, particularly Alzheimer's disease neuropathological change and vascular pathology. Increasing burdens of these pathologies are the major correlates of dementia, especially neurofibrillary tangles, but there is substantial overlap in pathology between those with and without dementia, particularly at intermediate burdens of pathology and also at the oldest ages. Furthermore, additional pathologies such as limbic‐predominant age‐related TDP‐43 encephalopathy, ageing‐related tau astrogliopathy and primary age‐related tauopathies contribute to late‐life dementia. Findings from ageing population‐representative studies have implications for the understanding of dementia pathology in the community. The high prevalence of pathology and variable relationship to dementia status has implications for disease definition and indicate a role for modulating factors on cognitive outcome. The complexity of late‐life dementia, with mixed pathologies, indicates a need for a better understanding of these processes across the life‐course to direct the best research for reducing risk in later life of avoidable clinical dementia syndromes.

## Graphical abstract

Population‐representative and community‐based neuropathology studies provide an opportunity to assess the spectrum and variation in ageing‐associated pathologies and their relationships to dementia. Alzheimer's and vascular pathologies are major associations of dementia, but there is overlap in pathology burden between individuals with and without dementia, especially at intermediate burdens of pathology and in the oldest old. Multiple pathologies combine to underlie dementia in the older population, including more recently defined pathologies, implying a need for better stratification and tailored therapy.

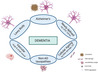

**Key points**

- ADNC and vascular pathology are highly prevalent in the older population and commonly coexist, with implications for diagnostic specificity and disease definitions.
- There is overlap in pathology burden between individuals with and without dementia, especially in the oldest old, with a lack of diagnostic thresholds.
- There is an important contribution to dementia from “non‐conventional” pathologies, including modulating and resilience factors and novel ageing‐related pathologies.
- Multiple pathologies combine to underlie dementia in the older population, implying a need for better stratification and tailored therapy.

## EPIDEMIOLOGICAL NEUROPATHOLOGY

CFAS is a longitudinal population representative study of cognitive impairment and frailty in the ageing (65 yrs and above UK) population.^1^, ^2^ Based on six urban and rural UK centres, individuals were recruited from general (family) practitioner registers, and in the UK all individuals should be so registered. Thus, recruitment to the study was based solely on being a member of this general older population and, in contrast to case–control studies, not on preselected clinical criteria. CFAS developed a brain donation cohort (donation rate 3% of full cohort), which has now accrued >550 brains, and is also population‐representative, although it is enriched for dementia.^3^, ^4^, ^5^

A population cohort design brings an epidemiological perspective that allows an unbiased assessment of the relationship between pathology and risk factors for dementia, without circular reasoning that may be inherent in clinically recruited or clinicopathologically defined groups. This also avoids ceiling‐floor effects due to selecting cases at extreme ends of a spectrum and includes the full spectrum and the full mixture of pathology in the population. A population‐representative cohort design is complementary to a case–control design, the latter having strengths in depth of characterisation and in its well‐defined groups that are ideal for addressing specific mechanisms and focused questions, whilst clinical information on population cohorts may be limited, suited to a large sampling approach. However, clinical trials and studies developed in volunteer cohorts in different clinical settings, including highly specialised tertiary clinics, show differences in demographics and education and have a higher representation of dementia in younger individuals (<80 yrs) compared to the general population, where most dementia is in the oldest old.^6^, ^7^, ^8^, ^9^, ^10^ Frequencies of different pathologies differ between clinic and community‐based cohorts, with more vascular disease and mixed pathology in community/population cohorts and more Lewy body, atypical and single pathologies in clinic‐based studies.^11^ Relationships of pathology burden to dementia, the clinical courses, and the severity of specific dementia‐related pathologies in individuals with dementia differ between these study types.^7^, ^11^ Case‐control studies may have an artificial dichotomy between controls selected to be very fit and strong clinical phenotypes (a ceiling‐floor effect), potentially representing ends of a spectrum for those pathologies where such a spectrum is present in the population. Furthermore, the inclusion of the pathology in the clinical definition inherent in preselection into clinicopathological diagnostic categories can introduce circular biases in understanding how specific pathologies are related to dementia. Consequently, there are considerable limitations in seeking to generalise from clinic‐based cohorts to the population, where there will be lower predictive values of specific pathologies for dementia and where mixed dementias are not examined. Thus clinical interventions and diagnostic approaches developed and tested in younger preselected cohorts may show different efficacy in the older populations in which most dementia occurs. Studies such as CFAS, with a population basis, are therefore essential to understand the pathological basis of dementia in a general late‐life population whilst biomarker and intervention studies need to take account of the nature of dementia in the oldest old.

A strength of population cohorts is the length of study and richness of accumulated data. CFAS has been accruing neuropathological donations for >30 yrs. Although it is based on the general United Kingdom population, this is within a particular timeframe so that for CFAS I, baseline interviews took place in the early 1990s. To examine temporal trends a second cohort, the CFAS II study, was developed with sampling in the same geographical areas as three of the MRC CFAS centres (called CFAS I), using as similar a methodology as possible. This identified substantial decline in dementia occurrence in the population, with a reduction in the age‐specific prevalence and incidence of dementia.^12^, ^13^ This significant trend was confirmed by a combined analysis of several studies across high‐income countries.^14^ However, a neuropathology cohort was not developed for CFAS II, so that the neuropathological basis of this change in dementia incidence has not been defined. The neuropathological studies in this review therefore all relate to MRC CFAS, henceforth referred to a CFAS.

The overall structure of CFAS, including the interview structure was previously described.^5^ Briefly, dementia status was determined in life from structured interviews to capture systematically information relevant to dementia diagnosis using a well‐validated algorithmic assessment (AGECAT, as part of the Geriatric Mental State Examination) supplemented at death by information from death certification and a retrospective informant interview (RINI). This approach is reliable as an assessment of dementia and is suitable for large‐scale studies,^15^ but lacks clinical information on putative dementia subtypes, and detailed physical and investigative testing, that are available in clinic‐based case–control series. More finely graded information on dementia status, however, is available, including mini‐mental state examination (MMSE) scores, Cambridge cognitive examination (CAMCOG),^16^ cognitive trajectory based on transitions through MMSE and data on mild cognitive impairment.^17^, ^18^ Measures for additional outcomes have also been development, including delirium and frailty.^19^, ^20^

The aim of this narrative review is to define those insights into the pathological basis of dementia that have come from this *Epidemiological Neuropathology* approach. The review mainly concerns conventional neuropathological lesions, reflecting neurodegenerative and vascular pathologies. Although focused primarily on CFAS, the context of findings from other cohort studies is also discussed (Table 1, and additional studies in^21^), but this is not a comprehensive review of the extensive findings from those studies. A population‐based neuropathology study may be defined as one where the sampling frame is a general population defined by geographical boundaries, where respondents are recruited from all subgroups of the selected population and where post‐mortem brain examination of respondents is sought across all cognitive states.^22^ There are several population‐representative neuropathology studies globally, some of which recruit from the general ageing population and others from specific demographic groups.^23^, ^24^, ^25^, ^26^, ^27^, ^28^, ^29^, ^30^, ^31^, ^32^ In addition, community‐based and large convenience cohorts, are important contributors in this area. These latter cohort types differ from population‐representative cohorts in that they do not have the unbiased recruitment strategy of population cohorts. However, their large sizes and lack of preselection into clinico‐pathological groups allow them to address similar questions to population‐representative studies.

**TABLE 1:** Examples of major population‐representative and community‐based neuropathology cohorts.

Study	Country	Age^b^	Population
CFAS	UK	65+	General UK rural and urban^a^
CC75C	UK	75+	General city population, Cambridge, UK^a^
Vantaa 85+	Finland	85+	General city population, Vantaa, South Finland^a^
Honolulu Asia Ageing Study (HAAS)	USA	70+	Japanese‐American men, Hawaii^a^
Hisayama	Japan	65+	Japanese subrural community^a^
Cache County Study	USA	65+	Rural community, Cache County, Utah^a^
Adult Changes in Thought (ACT)	USA	65+	Group Health Cooperative members, Seattle area
Rush Religious Orders Study (ROS)	USA	75+	Catholic nuns, priests and brothers community cohort
Rush Memory and Ageing Project (MAP)	USA	75+	Older persons without known dementia community cohort, Chicago, Illinois

^a^ Core information on these population‐based cohorts, see Zaccai et al.^22^

^b^ Age at recruitment.

## PREVALENCE OF CONVENTIONAL PATHOLOGIES AND RELATIONSHIP TO DEMENTIA

A key finding from CFAS and other cohort studies is the high prevalence of neurodegenerative and vascular pathologies in the ageing population with Alzheimer's disease neuropathological change (ADNC) and vascular pathology being major correlates of dementia. CFAS published two major studies reporting the overall burdens of pathology and its relationship to dementia. The first, published in 2001, was based on an initial data release from 209 donations (acquired up to July 1998), with an age range of 70 to 103 yrs and of whom 48% had dementia at death.^3^ This showed a high prevalence of ADNC, including diffuse and neuritic plaques and neurofibrillary tangles (NFT), assessed using a modified Consortium to Establish a Registry for Alzheimer's Disease (CERAD) approach,^33^ with only 13% of donations having a “normal” brain and 36% satisfying CERAD neuropathological criteria for Alzheimer's disease (AD). In this review, AD refers to a type of dementia associated with plaques, NFT and other lesions composed variably of Aβ and tau proteins found in brain tissue. ADNC refers to these lesions irrespective of the clinical state. Thus, ADNC is used here as a purely pathological term that may encompass those lesions found in AD, those in preclinical AD destined to progress to dementia, and similar lesions in individuals where there may have been no trajectory to progression. Vascular lesions, consisting of lobar infarcts, lacunar infarcts and/or small vessel disease (SVD) were also highly prevalent, occurring in 78% of all cases.^3^

ADNC was related to dementia, especially cortical NFTs. Multiple, but not single, vascular pathologies were over‐represented in those with dementia. Plaques and NFT improved the dementia model, particularly the presence of moderate to severe cortical NFT. Multiple vascular pathologies, low brain weight and severe cerebral amyloid angiopathy (CAA) were also related to dementia. The CAA finding reflects those in other population‐based studies, where prevalence rates for CAA are higher among older people with dementia than without, suggesting that it is an important contributor to dementia.^34^ Lewy bodies, present in 11%, were not related to dementia (as a dichotomised variable). The model predicted dementia status in 75% of cases but no individual feature was sufficient in relation to an individual.

**FIGURE 1:**
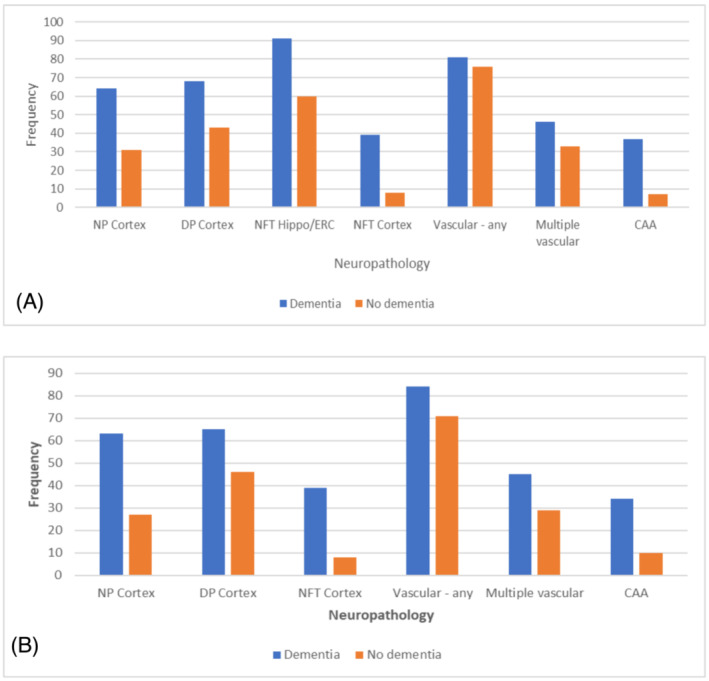
Comparison of frequency of pathological lesions in individuals with and without dementia in the; A. 2001 dataset (n = 209), B. 2009 dataset (n = 456). NP neuritic plaques, DP diffuse plaques, NFT neurofibrillary tangles, CAA cerebral amyloid angiopathy, hippo/ERC hippocampus and entorhinal cortex. For the Alzheimer‐type lesions, frequencies are for moderate to severe measures according to CERAD.

However, there was a high frequency of dementia‐related neuropathologies irrespective of whether dementia was present (Table 2, Figure 1A) with overlap, particularly for intermediate burdens of pathology. Thus, other factors are important in determining which individuals will show cognitive deterioration.

**TABLE 2:** Frequencies of neuropathology in the CFAS 2001 published data (n = 209).

Dementia status at death	Dementia %		No dementia %	
ADNC (CERAD score	Mod/severe	None	Mod/severe	None
Neuritic plaques ‐ neocortex	64	15	32	43
Diffuse plaques ‐ neocortex	68	20	43	31
NFT – hippocampus/entorhinal cortex	91	2	60	18
NFT ‐ neocortex	39	39	8	66
Vascular				
Any vascular lesion	81		76	
Multiple vascular	46		33	
Severe CAA	37		7	

Further data from CFAS, based on a larger cohort of 456 individuals and a mean age at death of 87 was published in 2009.^4^ Results supported and extended the previous findings, demonstrating high prevalence of ADNC and vascular pathologies, with NFT in the hippocampus and entorhinal cortex being the most prevalent pathology (92% of individuals). Moderate to severe NFT in neocortex was the best discriminator between individuals with and without dementia. This is consistent with other studies showing that NFT accumulation is highly associated with cognitive decline, and more so than amyloid.^35^ All individuals with severe neocortical NFTs had dementia so that this was a perfect predictor with an infinite odds ratio. But this lacks sensitivity as many individuals with dementia do not have NFT of this severity. Again, there was substantial overlap in pathology between individuals with and without dementia, similar to those in the earlier report (Table 3, Figure 1B). There were 3% of individuals who had substantial pathology without dementia whilst 15% of those with dementia had only modest or low pathology.

**TABLE 3:** Frequencies of neuropathology in the CFAS 2009 published dataset (n = 456).

Dementia status at death	Dementia %		No dementia %	
ADNC (CERAD score	Mod/severe	None	Mod/severe	None
Neuritic plaques ‐ neocortex	63	19	27	45
Diffuse plaques ‐ neocortex	65	9	46	30
NFT ‐ neocortex	41	33	4	63
Vascular				
Any vascular lesion	84		71	
Multiple vascular	45		29	
Severe CAA	34		10	

The larger cohort in the second paper was of sufficient size to estimate true population frequencies. Relationships to dementia were established using an attributable risk approach, which estimated relative contributions of specific pathologies to dementia at death. Contributors to dementia and their estimated contributions were; age (18%), small brain (12%), neocortical NFT (11%), neocortical neuritic plaques (8%), SVD (12%), multiple vascular pathology (9%), hippocampal atrophy (10%), CAA (7%) and Lewy bodies (3%). Together these factors explained 96% of the risk of dementia.

High prevalences of pathology in CFAS, particularly of ADNC and vascular pathologies, reflect those reported in other community‐based studies, with ADNC being the most prominent association with dementia, and with vascular measures (including microinfarcts and anterior cortical infarcts) and cortical Lewy bodies also being important associations. These studies also showed high burdens of pathology, sufficient for example for a diagnosis of AD, in individuals without dementia, although with more pathology in those with dementia, confirming the substantial overlap.^23^, ^24^, ^27^, ^29^, ^30^, ^31^ The importance of cortical NFT as an associate of dementia in both the first and second CFAS analyses reflects existing studies showing cortical NFT as the strongest pathological associate of dementia.^36^, ^37^

The neuropathology data collection in CFAS also included a range of other cellular pathologies including neuronal loss, gliosis, brainstem ADNC, pigmentary incontinence, granulovacuolar degeneration and Hirano bodies. Analysis of these in a combined cohort from the CFAS and CC75C^32^ studies (n = 638) showed that most of these variables had a significant association with dementia that was not attenuated by cortical neuritic plaques and Braak NFT stage. Five cases in this large cohort had Pick bodies and all of these had dementia.^38^ Hippocampal sclerosis, which is associated with TDP‐43 pathology, has also been shown to relate to dementia in the HAAS cohort.^27^ Thus, dementia in older age is associated with a broader range of lesions than those assessed and staged in the usual neuropathological diagnostic schemes.

In this context, it should be noted that some pathologies have not been well addressed to‐date in CFAS and other longitudinal studies. There is evidence that pathological changes of progressive supranuclear palsy and other atypical tauopathies are more widespread in community samples than previously recognised, with low sensitivity of the clinical diagnosis for the pathology.^39^, ^40^, ^41^ Argyrophilic grain disease has also been found to be common in a serial forensic and a community‐based cohort, associated with increasing age and with psychiatric and appetite disorders.^42^, ^43^ More thorough assessments of these and related pathologies in longitudinal cohorts are therefore warranted to understand their relative contributions to late‐life dementia in a population setting.

**FIGURE 2:**
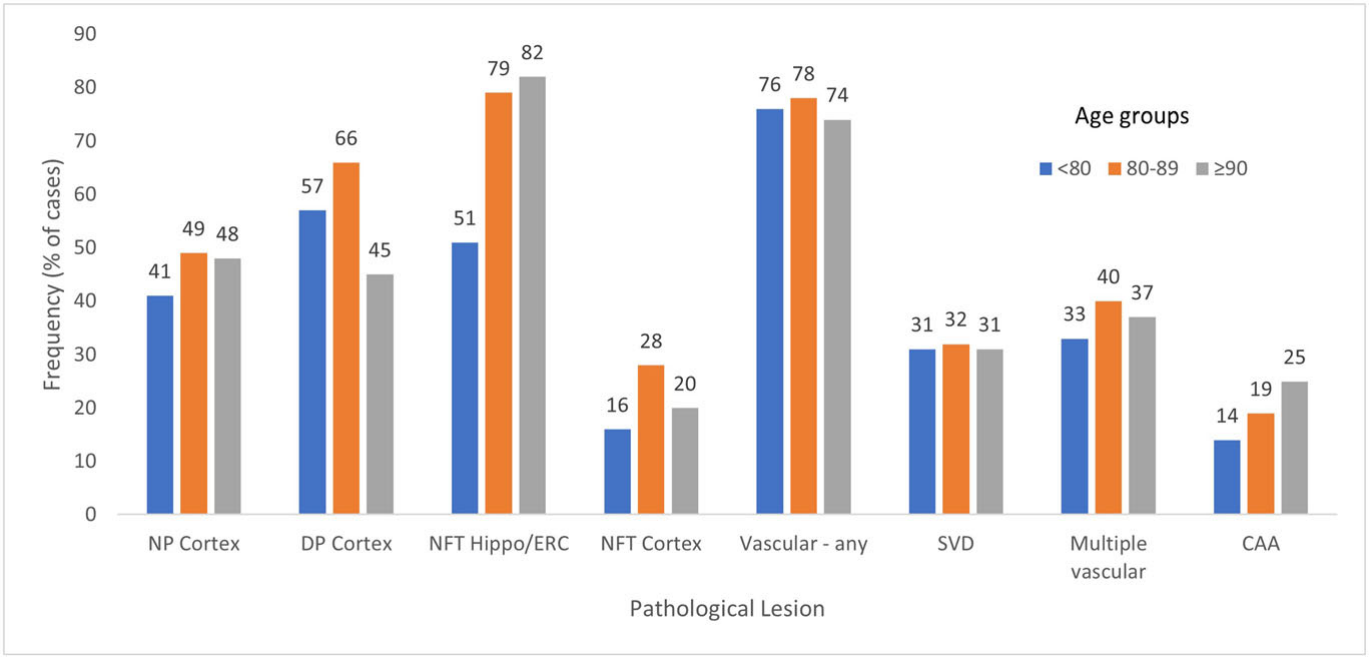
Frequencies of pathology for different pathology types across three age groups, <80 yrs, 80‐89 yrs, >90 yrs shown as separate colour bars. Data from the 2001 dataset. NP neuritic plaques, DP diffuse plaques, NFT neurofibrillary tangles, CAA cerebral amyloid angiopathy, hippo/ERC hippocampus and entorhinal cortex.

Pathological lesions showed no variation in their frequency across the four‐decade age‐span of the cohort in the 2001 CFAS dataset (Figure 2),^3^ although this is an area that would benefit from more detailed analysis in CFAS. This is despite epidemiological evidence demonstrating that dementia rises across the late life spectrum,^44^ which may imply a changing relationship of pathology to dementia. Similarly, the frequency of Lewy pathology does not show a change across the ageing spectrum.^45^ However, a more detailed analysis of tau pathology based on Braak NFT and BrainNet staging (see below) showed that stages increase with ageing amongst those without dementia,^46^ consistent with observations showing that tau pathology increases with age^47^; however, in CFAS this increase was not seen in those with dementia. Atherosclerosis and arteriolosclerosis have been shown to increase with age in ROSMAP, suggesting that vascular pathology may change with age.^48^

## STAGING SCHEMES BASED ON NEUROANATOMICAL HIERARCHY IN A POPULATION‐REPRESENTATIVE SETTING

### Staging for tau pathology

The major dementia‐associated pathologies show continuums of pathological burdens in the older population. Schemes for the staging of ADNC and Lewy body pathology reflect this continuum. Staging schemes for neurodegenerative pathologies are based on the anatomical progression of pathology and were defined on large convenience cohorts. These provide an approach that is distinct from the CERAD scheme, used for the 2001 and 2009 CFAS papers, that is based on densities of lesions in isocortex.^33^ Thus Braak and Braak NFT staging proposes a progression of NFT through entorhinal, then limbic and then isocortical regions.^49^, ^50^ Work in CFAS shows that Braak and Braak NFT staging reflects the hierarchy of pathological progression in the older population with individual donors falling along the stererotypical spectrum for progression.^46^ For tau pathology, Braak and Braak NFT staging also corresponded closely with BrainNet Europe (BNE) tau staging based on an assessment of neuropil threads,^51^ and with only 1% of individuals deviating from this staging scheme. Inclusion of either the Braak and Braak NFT or BNE tau staging had a similar effect in dementia models suggesting that the relationship to dementia is with neuronal tau in general and not specifically NFT or threads, and again considerable overlap in burdens of tau pathology was found between individuals with and without dementia. Tau pathology also shows a hierarchical pattern of spread through entorhinal/hippocampal structures that reflects neuroanatomical connectivity in this region and allows for the definition of a hippocampal stage.^52^ This also shows a relationship to dementia status and is highly correlated with Braak and Braak NFT stage, so that hippocampal staging does not provide additional information on dementia status when corrected for Braak and Braak NFT stage.

### Staging for Aβ pathology

Thal staging proposes phases for Aβ progression from isocortical through allocortical (entorhinal, hippocampal, cingulate), diencephalon/basal ganglia, brainstem and cerebellum.^53^ Agreement of Aβ pathology in CFAS with Thal phases was excellent, with only two individuals deviating from the scheme, so that Thal phase is applicable to the continuum of pathology in a population setting.^54^ CAA was also staged, based on the number of brain areas involved. This method of staging CAA was highly correlated with assessment of CAA based on severity of vascular involvement in cortical areas.^55^ CAA was present in around 75% of respondents in CFAS, and 49% were CAA type I (i.e. with capillary involvement). This prevalence of CAA is similar to that of 79% in the combined community‐based Religious Orders Study and Rush Memory and Ageing Project (ROSMAP),^56^ and higher than in an unselected autopsy series (54%)^57^ and in the Honolulu Asia Ageing Study (HAAS, males only) (44%).^58^ In ROSMAP, CAA showed an association with possession of either the ε4 or ε2 alleles of *APOE* and increased likelihood of CAA type 1.^59^ As in these other cohorts, occipital cortex was the most frequently involved region in CFAS. CAA also correlated with Thal phase. Thal phase, CAA and CERAD provide several different approaches to assessing Aβ‐pathology in the ageing brain; however, using both logistic regression and machine learning approaches, each of these parameters has similar effects on dementia models, possibly because they are highly correlated,^54^, ^60^ so that assessing different aspects of Aβ pathology does not improve dementia prediction over single measures.

### Staging for α‐synuclein pathology

Braak Lewy body staging proposes an ascending progression from medulla through pons, midbrain, transentorhinal/allocortex and finally to neocortical structures, a progression referred to as the Braak hypothesis.^61^ Dementia with Lewy Bodies (DLB) consensus criteria also classify cases into brainstem predominant, limbic and isocortical stages.^62^ The newer Lewy Pathology Consensus criteria are based on a dichotomous assessment of Lewy pathology in olfactory, amygdala, limbic and neocortical areas and have shown superiority in classification into distinct categories to Braak.^63^ This scheme also takes account of amygdala involvement, which has been associated with ADNC. A study based in two of the CFAS centres (n = 208) using immunohistochemistry to α‐synuclein found a frequency of Lewy bodies in any brain area of 39%, with an estimated population‐prevalence of 37%,^45^ which was more sensitive than H and E alone, which identified Lewy bodies in 11% of cases.^3^ Staging using immunohistochemistry found that 51% conformed to DLB consensus/Braak Lewy staging with 17% having an amygdala predominant pattern. Around 20% of individuals did not conform to those staging schemes. Thus, in contrast to the findings for Braak and Braak NFT and Thal Aβ schemes, the Braak Lewy and DLB consensus staging are good for Parkinson's and Dementia with Lewy Body diseases, for which they were developed, but do not capture the full spectrum of α‐synuclein/Lewy body pathology in an ageing population.

## SVD AS A CONTRIBUTOR TO DEMENTIA

**FIGURE 3:**
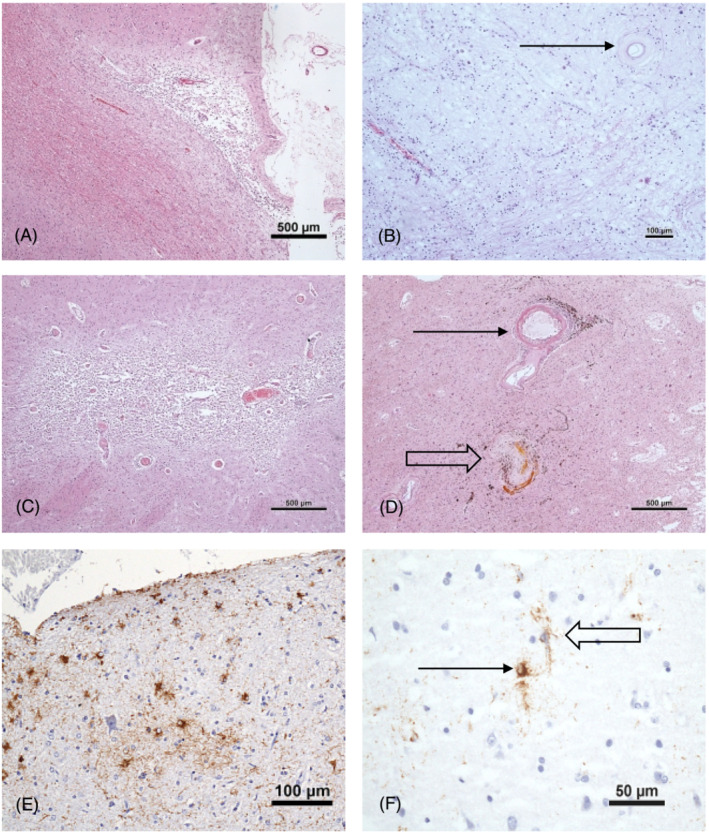
A. Microinfarct in parietal cortex. B. Severe white matter pallor and vessel with severe arteriolosclerosis, characterised by complete loss of smooth muscle cells (arrow). C. Lacunar infarct in basal ganglia containing numerous foamy macrophages. D. Complicated small vessel disease in basal ganglia; vessel showing lipohyalinosis with foamy macrophages (solid arrow) and sclerosed vessel with blood pigment representing “microhaemorrhage” (open arrow). E. Thorn‐shaped astrocytes, a component of ARTAG, in entorhinal subpial cortex. E. Astrocyte with finely granular tau temporal cortex (arrow). Tau‐positive astrocyte processes present around a capillary (open arrow).

Vascular pathology is highly prevalent in the late‐life population and an important contributor to dementia, particularly if multiple vascular pathologies are present.^3^, ^4^ Indeed, in CFAS, few dementia cases lacked both ADNC and vascular pathology and both pathology types were found to be common in individuals with and without dementia. SVD, defined in CFAS as any one of moderate/severe arteriosclerosis, lacunar infarcts, severe white matter lesions (WML) and microinfarcts (Figure 3A–D), appears to be more important as a risk factor for cognitive decline than major stroke.^64^ CAA may also be included within the scope of SVD (see below). Studies in ROSMAP suggest that arteriosclerosis is associated with atherosclerosis and that they contribute to AD clinical syndrome.^48^

WML, which can be divided into those in a deep subcortical and periventricular locations, can be identified on MRI scan and was assessed in CFAS using post‐mortem MRI of brain slices and semi‐quantified using a modified Schelten's score.^65^ WML are very common in older people^66^, ^67^ with relationships to dementia, depression and impaired mobility.^68^, ^69^, ^70^, ^71^ They are often considered to be caused by and are used as a radiological marker of, SVD.^72^ In CFAS, they were very frequent, with periventricular lesions in 95% of individuals with dementia and 87% of those without, and deep subcortical lesions in 73% of individuals with dementia and 60% of those without. WML were over‐represented in dementia suggesting that they are an independent contributor to dementia, but with a large overlap in burdens of WML between individuals with and without dementia.^4^ Studies of WML pathology, pathogenesis and transcriptomics in CFAS have been undertaken using post‐mortem MRI‐directed sampling^73^, ^74^, ^75^, ^76^, ^77^ and a population sampling approach^78^ and the findings have been reviewed elsewhere.^79^ Of note here, however, is that CFAS and other studies suggest that WML are not only a manifestation of arteriolosclerotic SVD, but that other factors, such as cortical neurodegeneration, ageing, CAA and other vascular mechanisms may also contribute,^78^, ^79^, ^80^, ^81^, ^82^ complicating the assumption that they are a biomarker for SVD.

### Microinfarcts

Microinfarcts are common in brain ageing.^83^ Thought to be ischaemic in origin, they are visualised on microscopic examination, although they may be visible macroscopically, often having a slit‐like pattern when in the cortex. There is no consensus definition and there are differences between radiological and pathological definitions.^84^ Microinfarcts in three CFAS subcohorts (n = 331) were assessed in nine cortical and subcortical areas.^85^ Microinfarcts were common, found in 36% of cases, comparable to a prevalence of 30% found in the Religious Orders Study.^86^ Microinfarcts in CFAS were associated with other manifestations of cerebrovascular pathology, including a history of stroke, higher deep WML score and history of hypertension. More cortical regions with microinfarcts were associated with dementia risk and more subcortical regions with microinfarcts with impaired mobility and falls, so that microinfarcts are relevant to these late‐life clinical syndromes. Cortical microinfarcts were associated with CAA and subcortical infarcts with arteriolosclerosis in the ROSMAP Study,^87^ but a relationship of microinfarcts to CAA was not demonstrated in CFAS.^54^

The association of microinfarcts with dementia has been demonstrated in multiple other cohorts, and microinfarcts were found to be important in clinically expressed SVD.^27^, ^29^, ^88^, ^89^, ^90^ However, in CFAS the addition of microinfarcts did not improve the predictive power of SVD diagnosis.^85^ Rather, WML were particularly related to dementia whilst microinfarcts were associated with impaired mobility and falls. Overall, assessing SVD using a constellation of pathological features seems to be a valid approach. This study suggested that, whilst assessing the parenchymal manifestations of SVD (WML, lacunes and microinfarcts) may be important, assessing the direct vascular damage, namely arteriolosclerosis, may add little to the assessment of SVD (though this differs from the implications of the VCING criteria, see below).

### Limitations of post‐mortem assessment of vascular neuropathology

Assessment of vascular pathology in post‐mortem brains is limited by the lack of standardised criteria with the stereotypy and reproducibility of the staging schemes used for the neurodegenerative proteinopathies.^91^ Vascular pathology may be assessed qualitatively by classifying the type of vascular neuropathology; I large or cortical infarcts; II multiple small or lacunes; III strategic infarcts; IV hypoperfusive lesions; V cerebral haemorrhages; VI cerebrovascular disease with AD.^92^ Staging approaches have been developed, such as that of Deramecourt et al, based on the evaluation of arteriolosclerosis, CAA, perivascular changes, myelin loss, cortical microinfarcts and infarcts in frontal and temporal lobes, hippocampus and basal ganglia.^93^ The more recent Vascular Cognitive Impairment Neuropathology Guidelines (VCING) criteria were developed following a Delphi‐type consultation phase and empirical testing of criteria in brain donations from individuals without significant neurodegenerative disease. This approach yielded a scoring scheme for vascular pathology based on the presence/absence of moderate/severe occipital leptomeningeal CAA, moderate/severe arteriolosclerosis in occipital white matter and at least one large infarct.^94^ Assessment of SVD markers is important in these schemes. The development of staging schemes has not yet taken into account data from population‐representative cohorts. VCING has been used in small cohorts,^95^, ^96^ but these schemes have not yet been tested for their ability to quantify vascular pathology and for their predictive power for dementia in a general ageing population setting.

## LACK OF THRESHOLDS FOR CONVENTIONAL PATHOLOGIES IN IDENTIFYING DEMENTIA

**FIGURE 4:**
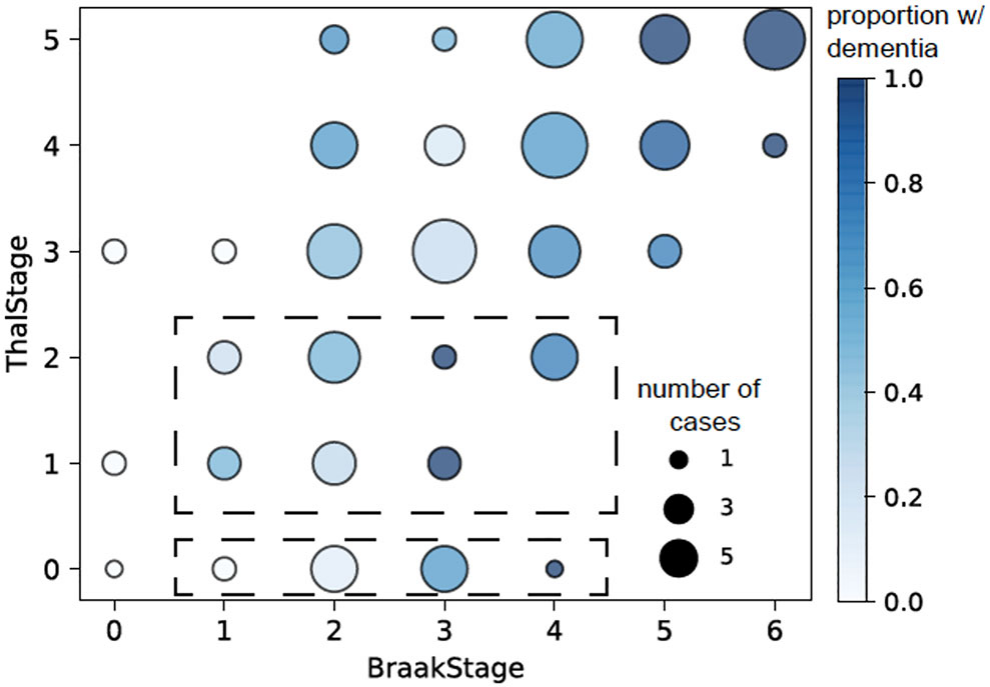
Scatterplot of Thal Aβ phase vs Braak and Braak NFT stage allowing identification of variation in relationships of these measures. Areas corresponding to PART‐definite and –possible are outlined with dotted lines. Reproduced from Wharton et al, Acta Neuropathol Commun 2019; 7:198.

The classical neurodegenerative and vascular pathologies have important associations with dementia (see above). For ADNC, NFT are particularly associated with dementia and there is an increasing proportion of individuals with dementia with increasing Braak and Braak NFT stage and Thal phase (Figure 4 and^54^). Thus, in CFAS, virtually all individuals with Braak and Braak NFT stage VI showed dementia at death. However, many individuals with dementia at death do not have such high burdens of pathology. Furthermore, the main pathologies observed in the ageing brain and associated with dementia show continuums of severity and overlaps in burdens between individuals with and without dementia, which is seen particularly at intermediate burdens of pathology.^3^, ^4^ A corollary of this is the lack of thresholds for dementia, which implies that it is unlikely to be able to establish clear, and highly accurate, cut‐offs for the pathological definition of dementia. This is reflected in current neuropathological diagnostic approaches to dementia, such as the CERAD and ABC systems for Alzheimer's disease, which are focused on determining if AD is the cause of dementia in the situation of known dementia.^33^, ^97^ The incorporation of both clinical and pathological information into neuropathological disease definition is useful for practical case assessment in a clinical setting but gives rise to circular reasoning in identifying pathological markers of dementia. For ADNC, whilst the likelihood of dementia increases with increasing burden of pathology there are individuals with low Thal phase and Braak and Braak NFT stage who nevertheless have expressed dementia during life. At the other end of the spectrum, whilst Braak and Braak NFT stage VI pathology is virtually always associated with dementia, there remain individuals with high burdens of ADNC who have not expressed dementia within the neocortical stage.^46^, ^54^ Furthermore, the relationship between ADNC and dementia status is attenuated at the oldest ages so that whilst NFT and plaques distinguish individuals with and without dementia in the younger old (60s and 70s), there is a convergence in the oldest old (80s and 90s).^98^

A potential limitation here in population studies is the binary assessment of dementia. In CFAS this is based on an algorithmic approach along with death certificate information and the RINI, which is reliable for this population setting. However, this does not capture some of the subtleties of dementia, nor early and intermediate states, such as mild cognitive impairment (MCI), which are likely to contribute to the variation in association of pathology to dementia. A further caveat is that MCI may be defined in different ways that may differ between population‐based and clinical studies, whilst the neuropathological profile of MCI is complex in a population setting.^99^, ^100^ It is also possible that some of the apparent resilience in autopsy cohorts, whereby there are individuals with significant pathology without dementia, could be accounted for by longer intervals between dementia testing and death^101^ and it is important to assess such potential artefactual influences. In CFAS, this was addressed by incorporating the information from death certificates and the RINI, but there were some individuals where dementia status remained “unknown” because of insufficient information to assign a state with certainty (30/456 individuals in the 2009 data release^4^). Apparent resilience may also be overstated when a binary dementia assessment is used and may be reduced if a continuous or categorical measure of cognition prior to death were used. This approach, however, would still face problems related to missing data, interval between last assessment and death, and the lower accuracy in assigning individuals to intermediate states.

In contrast to ADNC, brain atrophy continues to separate individuals with dementia from those without across the late‐life spectrum.^98^ Atrophy has also been shown to relate to dementia in ROSMAP.^31^ In HAAS, atrophy was associated with ADNC and microinfarcts but was also seen in those without pathological lesions and was independently associated with reduced cognition, even when accounting for education.^27^ Understanding the pathological basis of atrophy is therefore an important outstanding research question.

The existence of *low‐pathology dementia* cases is reflected in other studies that have identified individuals with clinical AD but who have “insufficient AD neuropathological change” at autopsy and where conventional pathological lesions do not explain all of cognitive decline.^102^, ^103^ The existence of *high pathology non‐dementia cases* is supported by studies finding “high pathology controls” particularly in very old people, with similar plaque burdens between individuals with and without dementia at death.^104^, ^105^

### Potential roles of additional processes in modulating dementia outcomes

These findings imply the existence of other contributory factors to dementia and roles for modulating agents and resilience factors. Some of this may be related to variations in co‐pathologies, including more recently defined pathologies (see below) and in cerebrovascular pathology,^101^, ^106^ but there are also multiple candidate factors in addition to classical pathologies such as cellular ageing mechanisms, neuroinflammation, and social and educational factors beyond the scope of this review. Notably, factors such as age and atrophy or brain weight are also significant contributors to dementia in models,^4^ and atrophy remains a discriminator between individuals with and without dementia across the late‐life spectrum, in contrast to ADNC.^98^

Ageing affects multiple cell processes and is associated with DNA damage and senescence.^107^ Oxidative stress and DNA damage have been implicated in AD and various neurodegenerative diseases, and in CFAS were found early in the progression of ADNC in both astrocytes and neurons.^108^ Examination of a subcohort within CFAS characterised by low Braak and Braak NFT stage (0‐II) showed that higher markers of DNA damage response were associated with lower cognition and gene expression studies showed this was accompanied by changes in pathways, such as cholesterol biosynthesis and signalling, that are relevant to neurodegeneration.^109^, ^110^ DNA and oxidative damage are also features of age‐associated white matter lesions and white matter surrounding lesions.^111^ DNA damage processes, either independently or interacting with other pathologies, may be another contributor to cognitive impairment.

Neuroinflammation, characterised by microglial and astrocytic responses, is associated with ageing and has also been implicated in dementia pathology. Studies using PET have suggested that microglial response may be early in AD progression, correlates with Aβ and tau pathology, and may switch from a protective role early in AD progression to a more pro‐inflammatory role at later stages.^112^, ^113^, ^114^ Microglial phenotypes are complex and assessment in tissue, particularly formalin‐fixed tissue, is limited by available markers.^115^ Longitudinal assessment is also not possible. Using available markers in CFAS, dementia was associated with higher CD68 and lower Iba1 suggesting that dementia is associated with a change in microglial phenotype.^116^

Astrocyte responses were also assessed in CFAS. Immunohistochemical studies suggested that astrocyte reactivity, measured by expression of glial fibrillary acidic protein, increases early with ADNC progression, associated with loss of the glutamate transporter EAAT2 and changes in the astrocyte transcriptome.^117^, ^118^ PET imaging studies also suggest that astrocyte responses are early in ADNC progression and may be protective.^119^ GFAP expression, measured in cortex using ELISA, was not an independent predictor of dementia in the CFAS cohort,^120^ whereas, in contrast, cortical astrogliosis did show a relationship to dementia in the HAAS cohort, which used a larger number of donations and more brain areas.^121^ Taking results of studies such as these together suggests that astrocytes may modulate responses to ADNC and other degenerative pathologies and may be a potential therapeutic target.

Other molecular species within classical lesions may also affect outcomes. Serum amyloid P component is a constituent of all types of amyloid, and can be demonstrated in Aβ deposits and neurofibrillary pathology.^122^ Brain serum amyloid P content, measured biochemically, is higher in those with dementia, independent of Aβ and tau, suggesting that this key amyloid component is a modulating factor for dementia and may itself be a therapeutic target.^123^

## SIGNIFICANCE OF HIGH PATHOLOGY PREVALENCE AND LACK OF THRESHOLDS FOR DISEASE DEFINITION

The high prevalence of the various dementia‐associated neuropathological lesions is highly relevant to current ideas on the definitions of disease. AD is currently defined as dementia associated with Alzheimer‐type neuropathological change. However, the identification of ADNC in cognitively intact individuals implies pre‐clinical disease, the importance of which has been elevated by the ability to detect ADNC in‐life through imaging^124^, ^125^ and CSF biomarkers,^126^ and by the therapeutic drive to treat early despite the only study with reduction in cognitive decline observed with medication was at a level that had a priori been deemed as not clinically significant.^127^, ^128^ Indeed, a biological definition of AD for research purposes, the ATN system based on Aβ, tau and neurodegenerative biomarkers, has been proposed by the National Institute on Ageing and Alzheimer's Association (NIA‐AA), which defines AD by the presence of pathological markers,^129^ and which has provoked debate into its use in practice.^130^ However, if the prevalence of ADNC in the over 65 s approaches 100% as they age, and where some individuals at death tolerate high burdens of ADNC without dementia, a definition of AD based solely on the presence of ADNC or AD biomarkers will lead to over‐diagnosis, especially as biomarkers increase in sensitivity. This may particularly be the case in the oldest old, where burdens of pathology are highest and relationships of pathology to dementia most attenuated. A key research question is to identify those factors that will predict progression so that treatment is appropriately targeted and to ensure that such markers remain relevant in the oldest age groups, where dementia is most common.

## DEVELOPING CONCEPTS OF NOVEL PATHOLOGIES

The last decade has seen the definition of several novel pathologies in the ageing brain, particularly in the oldest old, based on the application of immunohistochemistry to proteins that are already well‐characterised constituents of conventional pathologies.

### Primary age‐related tauopathy (PART)

Neuropathologists have long recognised cases, especially in the oldest‐old, with significant tau pathology in mesial temporal structures but with little or no amyloid. The use of Thal and Braak and Braak staging for Aβ and tau pathology respectively has allowed a better definition of these cases as primary age‐related tauopathy (PART). PART‐definite cases have been defined as Braak and Braak NFT stage I‐IV/Thal phase 0 and PART‐possible as Braak and Braak NFT stage I‐IV/Thal phase I‐II.^131^ Although there is some debate about whether PART is a distinct entity or represents variation within ADNC,^132^ the use of anatomical staging allows visualisation of pathological subgroups within the population that may have pathological and clinical significance. Other such variations also exist within ADNC: thus, there are individuals showing high Thal phase but low CAA stage and vice versa, reflecting variation in the patterns of deposition of Aβ. In CFAS data, PART‐definite showed a population frequency of about 10%.^54^ A scatterplot of Thal phase vs Braak and Braak NFT stage, which allows visualisation of these staging relationships, does not show a distinct separation of PART from the main sequence of the Aβ and NFT relationship (Figure 4). Furthermore, CFAS data did not demonstrate that those with PART had higher levels of dementia than non‐PART cases, although the comparison group here had Aβ pathology. Assessment in relation to a no‐pathology group was not performed and, whilst this may be ideal, individuals with no pathology are very rare and, indeed, what is called “normal” is very atypical in these population studies. In a population setting those with minimal pathology as defined by Braak and Braak NFT stages I‐II, which in many case–control studies would be defined as controls, in fact, fall within the PART group. This complicates the assessment of the real impact of dementia. PART also appears to show a relationship to another age‐related tauopathy, ARTAG (see below), so that it may be part of a constellation of late‐age tau pathology.

### Ageing‐related tau Astrogliopathy (ARTAG)

Tau, identified by antibodies such as AT8 (which recognised phosphorylation at serine 202 and threonine 205), may be found in astrocytes in brain ageing (Figure 3E,F). These may be subpial, subependymal or perivascular in location and are particularly prevalent in mesial temporal structures. A range of morphologies has been identified including thorn‐shaped astrocytes and fuzzy astrocytes. The tau stains particularly for 4R‐isoforms and there is staining with the Gallyas silver method suggesting that some of it is fibrillar. Thorn‐shaped astrocytes were found in nearly half of the CFAS cohort, most commonly in mesial temporal lobe but their inclusion in dementia models did not improve prediction.^46^, ^133^ Work in several cohorts has now led to the development of the diagnostic concept of ageing‐related tau astrogliopathy (ARTAG), and of consensus criteria for its identification. This is characterised by tau‐immunoreactive thorn‐shaped astrocytes at the glial limitans and white matter, and granular fuzzy astrocytes in grey matter that are distinct from those in other primary tauopathies.^134^, ^135^ In CFAS, tau‐positive thorn‐shaped astrocytes, a key component of ARTAG, did not improve dementia prediction.^46^ In the 90 + Study, cortical stage, but not limbic or brainstem stages, of ARTAG showed a relationship to dementia in the oldest old cohort.^106^ ARTAG expands the range of glial tau pathology and late‐life protein pathologies. Further work is required to define its roles in dementia and brain ageing.

### Limbic‐predominant age‐related TDP‐43 encephalopathy

Transactive response DNA‐binding protein of 34 kDA (TDP‐43) has been identified as the key protein in the proteinaceous inclusions that are present in motor neurons in MND/ALS and in frontotemporal and hippocampal areas in a subset of frontotemporal lobar degeneration that is characterised by TDP‐43 pathology (FTLD‐TDP).^136^, ^137^ Demonstration of the inclusions by immunohistochemistry is central to neuropathological diagnosis in these disorders, where inclusions are associated with nuclear mis‐localisation. TDP‐43 pathology has also been found in mesial temporal structures in brains in the oldest age groups, particularly in amygdala and hippocampus, but also cortical structures, where they have been associated with AD and hippocampal sclerosis.^138^ This has led to the diagnostic concept of limbic‐predominant age‐related TDP‐43 encephalopathy (LATE), defined by TDP‐43 pathology in mesial temporal structures. Occurring in older individuals, LATE involves amygdala, hippocampus and frontal cortex in a hierarchical manner and appears distinct from FTLD‐TDP.^139^ Assessment criteria have recently been proposed for LATE.^140^ Hippocampal TDP‐43 pathology was identified in 33% of a combined cohort (n = 642) from the CFAS and CC75C studies and those with inclusions were older.^141^ This is similar to the prevalence of 36% in an over‐90s cohort.^106^ TDP‐43 pathology increases with age, is associated with cognitive impairment and hippocampal sclerosis, and shows a relationship to ADNC that may vary in different cohorts.^142^, ^143^ Combining data from 13 population and community‐based neuropathology studies (including CFAS and CC75C), which included >5600 individuals with known cognitive status, has enabled a more accurate assessment of LATE. This approach revealed a prevalence of nearly 40% of donated brains in these late‐life cohorts. LATE is associated with ADNC, increasing with Braak and Braak NFT stage, but LATE can exist independently and appears to be an impactful contributor to late‐life dementia.^21^

## MOST LATE‐LIFE DEMENTIA IS ASSOCIATED WITH MULTIPLE PATHOLOGIES

**FIGURE 5:**
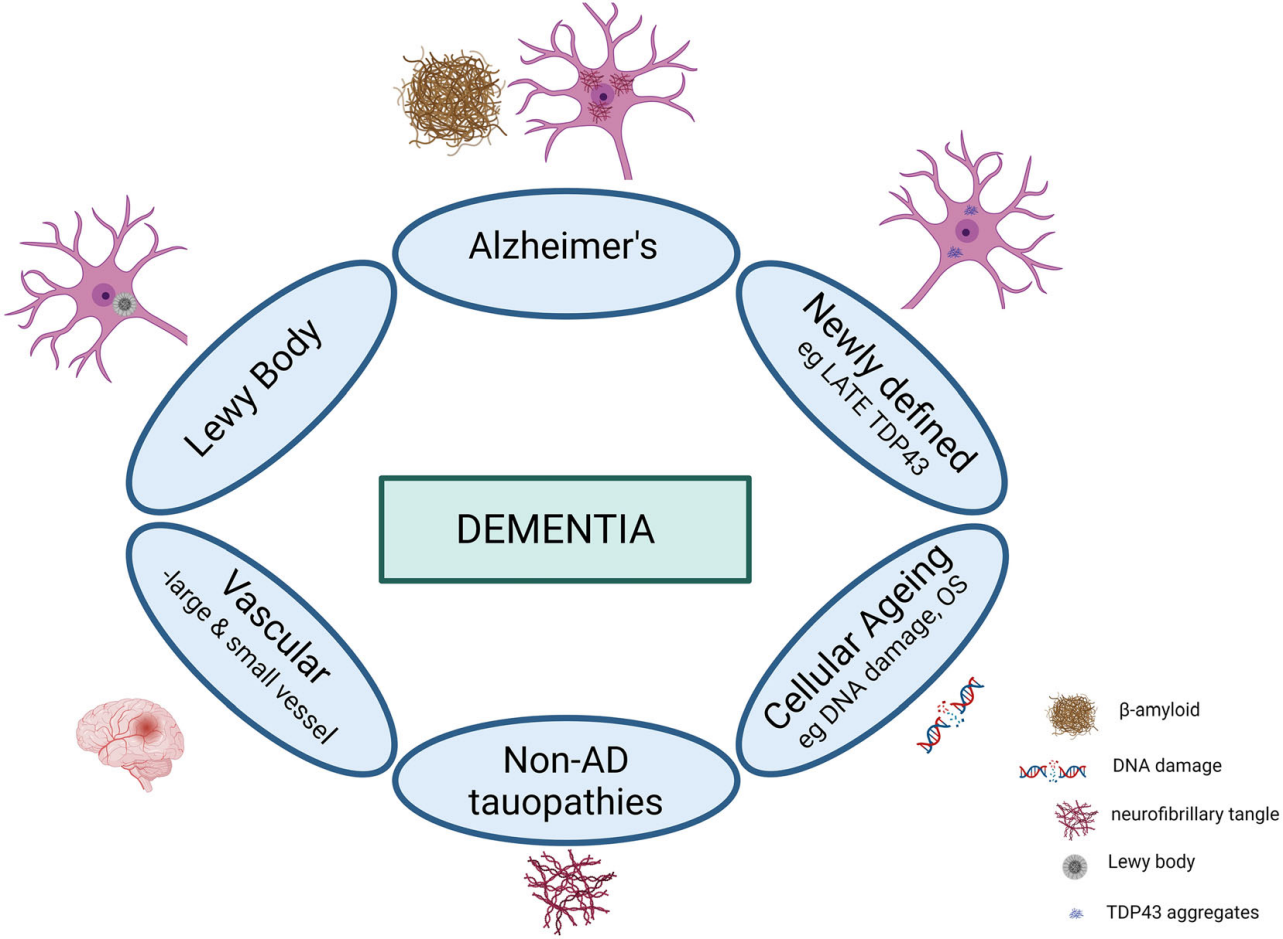
Multiple pathologies, including neurodegenerative, vascular and ageing mechanisms, commonly coexist in the elderly and can combine to contribute to dementia. Modified from Rahimi and Kovacs 2014. Created with BioRender.com

Multiple pathologies, especially the combination of ADNC and vascular pathology, determine the overall burden of dementia in late‐life in CFAS^3^, ^4^ and in other community‐based cohorts.^24^, ^29^, ^31^, ^101^ The presence of additional pathologies may lower the burdens of ADNC required for dementia to occur.^144^ The contribution of multiple pathologies suggests a potentially complex basis for the mixed dementias, including the newer entities such as LATE,^145^ and to this might be added ageing‐related cellular changes such as DNA damage, senescence and neuroinflammation (Figure 5). These pathologies may arise independently with additive effects, but pathologies may also interact and modelling interactions will be important for understanding lesion interactions and potential common mechanisms.^37^, ^146^ Thus, for example, AD and vascular dementia share risk factors and mechanisms, whilst vascular changes may contribute to the pathogenesis of AD.^147^, ^148^, ^149^, ^150^, ^151^ Further, the presence of higher burdens of Aβ and tau pathology increases the link between the vascular pathologies themselves and consequent microvascular parenchymal injury, suggesting an interaction between ADNC and microvascular pathologies.^152^ This has a number of implications for dementia. The common mixed dementia seen in an older population sample differs from that in younger clinic‐based samples and suggests that therapy targeted to a specific pathology may be less effective in the majority with mixed disease. Such studies emphasise the need for improved biomarkers to allow better identification of pathological processes in‐life and improved stratification for treatment.

## STUDY DESIGN

### Missing data

Utilising CFAS study design and robust methods of handling missing data makes it possible to estimate measures such as risk, prevalence and incidence of cognitive decline and dementia representative of the wider population. CFAS is designed to be the most robust population base for sampling by age group for epidemiological studies in the UK. In both CFAS I and II studies, random sampling was used to recruit at least 2500 participants in each centre, stratified by age group and oversampling to account for losses.^13^

Inverse probability weighting is used to adjust for both non‐response in CFAS I. Weights are calculated by birth cohort and sex, factors which are known for the entire population approached in CFAS, including participants who declined to take part, with resulting estimates being adjusted to represent the complete population. Many population studies using a two‐stage design do not take into account and adjust for design features, resulting in over‐optimistic confidence intervals for estimates.^153^ In CFAS estimates account for this using inverse probability weighting.^13^

An example where these techniques have been implemented in the CFAS neuropathology data was the estimation of Population Attributable Risk of dementia pathologies.^4^ In this study multiple imputation using chained equations was used to conduct sensitivity analyses. By imputing data for all individuals for unknown pathologies the robustness of models before imputation can be compared to the same model after for differences due to missing data. Effects of bias from sampling for the brain cohort were also addressed in this study using inverse probability weighting. The brain cohort was an older sample of the remaining baseline who died during the study period. As these characteristics were known, a sensitivity analysis could be back‐weighted to account for sampling effects.

The combination of study design for a truly representative population and the use of statistical methods of handling missing data makes it possible to make population‐representative estimates of epidemiological dementia pathology.

### Limitations of the epidemiological neuropathology approach

Cohort neuropathology studies such as CFAS provide a valuable perspective on late‐life dementia pathology. They have general limitations of post‐mortem neuropathology studies in that the tissue is taken at the end of life, although they do allow the study of pathology in relation to progression through pathological stages. Tissue is collected often over many decades but there may be issues with tissue quality, use of historical staging schemes which may be out‐dated and variability in neuropathological assessment, which can be dealt with by on‐going reassessments in the light of advances in staging and detection methods. Many studies, including CFAS, may have limitations in neuropsychological data due to lack of testing close to death. There may also be a lack of in‐life imaging and linkage to biomarkers in life, though limited availability of blood and CSF samples may allow some biochemical biomarkers to be assessed. An important goal in dementia research is defining markers not only of dementia status but which can predict progression and so might be useful to target early therapies. Autopsy studies have a potential role here by identifying candidate markers associated with dementia or shown to be modulating factors that could lead to in‐life biomarkers. New analytical approaches may also improve utility. Most assessments in CFAS have been related to a binary classification of dementia at death, but assessment of additional measures such as MMSE and cognitive trajectory may provide finer assessments and identification of factors affecting the rate of cognitive decline, albeit retrospectively.^103^, ^154^

A further issue relates to cohort size which may limit power for assessment of factors with smaller effect sizes. A potential approach here is combining data from multiple studies. For example, data from CFAS, CC75C and Vantaa 85 + have been combined in the EClipSE collaboration.^20^, ^38^, ^155^, ^156^ Recently Nelson et al combined data from 13 cohorts in an assessment of LATE in the population to obtain a fuller picture of the prevalence of TDP‐43 pathology and its relationships to ADNC and dementia.^21^ Such combinations require extensive efforts at harmonisation of data cohorts, which may only be suitable for more basic measures. A recent study using data from six postmortem community‐based cohorts (including CFAS and CC75C) found that harmonisation of measures is feasible, particularly for ADNC measures which could be compared with high confidence, whilst vascular measures were low to moderate confidence, reflecting the less well‐defined criteria for vascular pathology.^157^ This study also highlighted the complexity and multimorbidity underlying late‐life dementia. Although inter‐study harmonisation is difficult and limited for some pathologies, this is a potentially powerful approach to obtain greater power for analyses and a truer understanding of how pathologies relate to dementia.

Several of the population studies are based on very specific subgroups that may not be generalisable to the whole population. Although CFAS is representative of the over‐65 UK population, this is within a particular temporal framework. A further study, CFAS II, based in three of the same geographic areas but with an interval of two decades from the original CFAS cohort, showed lower age‐specific prevalence and incidence rates for dementia.^12^, ^13^ Whether this is reflected in changes in the pathological basis of dementia in this population is unknown. A recent birth cohort trend analysis in the ROSMAP cohort suggested that ADNC, LATE and Lewy body measures remain constant overall (though with some variation in Aβ and NFT measures), whilst atherosclerosis and arteriolosclerosis reduce across birth cohorts.^158^ Thus, change in brain vascular disease is a candidate for changes in dementia across time. Furthermore, some sectors of the population, including some ethnic minority groups, may be under‐represented in such cohorts. Comparison of findings between different cohorts is therefore important to identify the most generalisable findings.

## CONCLUSIONS

Conventional pathologies are highly prevalent in the ageing brain and most dementia in late‐life is due to mixed pathology. The lack of thresholds for dementia prediction and the lack of a deterministic relationship between conventional pathologies and dementia, particularly in the oldest old where most dementia in the population occurs, implies other contributory and resilience factors. Hence dementia is highly complex and its basis may change across the ageing spectrum so that better in‐life diagnostic biomarkers are required and better stratification for treatment and trials. This needs to consider novel pathologies, such as the TDP‐43‐associated LATE, and non‐conventional factors such as neuroinflammation and cell‐ageing mechanisms. Quantification of some pathologies in the ageing brain, particularly vascular pathology and cell‐ageing‐related pathologies, currently lack good operationalised criteria for assessment and reproducible schemes would improve the value of their measures in modelling. Individual cohort studies provide complementary insights into dementia, but attempts to combine and harmonise data is a valuable goal because it can provide a clearer picture of the pathological basis of dementia better generalised to the population.

## AUTHOR CONTRIBUTIONS

SBW – conceived the review and produced the first draft. All authors – contributed to the development and final draft of the manuscript, including figures.

## CONFLICT OF INTEREST STATEMENT

There are no conflicts of interest.

### PEER REVIEW

The peer review history for this article is available at https://www.webofscience.com/api/gateway/wos/peer-review/10.1111/nan.12923.

## ETHICS STATEMENT

All studies in CFAS have received ethical approval.

## Data Availability

The data that support the findings of this study are available on request from the corresponding author. The data are not publicly available due to privacy or ethical restrictions.
